# Clinical review: intensive care unit acquired weakness

**DOI:** 10.1186/s13054-015-0993-7

**Published:** 2015-08-05

**Authors:** Greet Hermans, Greet Van den Berghe

**Affiliations:** Laboratory of Intensive Care Medicine, Division of Cellular and Molecular Medicine, KU Leuven, Herestraat 49, B-3000 Leuven, Belgium; Medical Intensive Care Unit, Department of General Internal Medicine, University Hospitals Leuven, Herestraat 49, B-3000 Leuven, Belgium; Department of Intensive Care Medicine, University Hospitals Leuven, Herestraat 49, B-3000 Leuven, Belgium

## Abstract

A substantial number of patients admitted to the ICU because of an acute illness, complicated surgery, severe trauma, or burn injury will develop a de novo form of muscle weakness during the ICU stay that is referred to as “intensive care unit acquired weakness” (ICUAW). This ICUAW evoked by critical illness can be due to axonal neuropathy, primary myopathy, or both. Underlying pathophysiological mechanisms comprise microvascular, electrical, metabolic, and bioenergetic alterations, interacting in a complex way and culminating in loss of muscle strength and/or muscle atrophy. ICUAW is typically symmetrical and affects predominantly proximal limb muscles and respiratory muscles, whereas facial and ocular muscles are often spared. The main risk factors for ICUAW include high severity of illness upon admission, sepsis, multiple organ failure, prolonged immobilization, and hyperglycemia, and also older patients have a higher risk. The role of corticosteroids and neuromuscular blocking agents remains unclear. ICUAW is diagnosed in awake and cooperative patients by bedside manual testing of muscle strength and the severity is scored by the Medical Research Council sum score. In cases of atypical clinical presentation or evolution, additional electrophysiological testing may be required for differential diagnosis. The cornerstones of prevention are aggressive treatment of sepsis, early mobilization, preventing hyperglycemia with insulin, and avoiding the use parenteral nutrition during the first week of critical illness. Weak patients clearly have worse acute outcomes and consume more healthcare resources. Recovery usually occurs within weeks or months, although it may be incomplete with weakness persisting up to 2 years after ICU discharge. Prognosis appears compromised when the cause of ICUAW involves critical illness polyneuropathy, whereas isolated critical illness myopathy may have a better prognosis. In addition, ICUAW has shown to contribute to the risk of 1-year mortality. Future research should focus on new preventive and/or therapeutic strategies for this detrimental complication of critical illness and on clarifying how ICUAW contributes to poor longer-term prognosis.

## Introduction

Generalized muscle weakness, which develops during the course of an ICU admission and for which no other cause can be identified besides the acute illness or its treatment, is labeled “intensive care unit acquired weakness” (ICUAW). ICUAW may affect peripheral as well as respiratory muscles. The “loss of flesh and strength” in patients with life-threatening infections was described in the nineteenth century [1]. However, it took another century before it was understood that ICUAW can be evoked either by critical illness polyneuropathy (CIP) [2], by critical illness myopathy (CIM) [3], or by both [4] during the course of critical illness. ICUAW is a frequent complication of critical illness and is associated with a high morbidity and mortality of acute critical illnesses. In addition, recent data revealed that ICUAW may also have longer-term consequences, beyond the hospitalization phase. For example, ICUAW may be an important contributor to the post intensive care syndrome (PICS) [5]. This term includes the physical, mental, and cognitive dysfunctions that are part of the persisting disabilities, which extend beyond the acute hospitalization and have major impact on the quality of life of the growing population of ICU survivors.

This review aims to update the available knowledge regarding ICUAW, its clinical characteristics and diagnostic properties, the underlying mechanisms and predisposing factors, as well as its medical and socioeconomic consequences. In addition, possible avenues towards prevention and treatment of ICUAW will be reviewed.

## Incidence

ICUAW is a frequent problem. The reported incidence varies depending on the patient population studied and on the timing of the evaluation. Weakness at awakening was found to be present in 26–65 % of patients who were mechanically ventilated for 5–7 days, respectively [6, 7], and 25 % of these remained weak for at least another 7 days after awakening [8]. Among long-term ventilated patients (≥10 days) ICUAW was diagnosed in up to 67 % [9]. In another study 11 % of patients treated in the ICU for at least 24 hours developed ICUAW [10], and when the ICU stay increased to 7–10 days ICUAW at awakening was present in 24–55 % of patients [10–12]. In patients suffering from acute respiratory distress syndrome (ARDS), an ICUAW incidence of 60 % has been reported at the time of awakening [13], and at hospital discharge this incidence was still 36 % [14].

## Pathophysiology

The mechanisms underlying ICUAW are complex and involve functional and structural alterations in both the muscles and the nerves.

In CIP, the pathological finding is axonal degeneration [15]. The pathogenesis of such axonal degeneration remains incompletely understood, in part explained by the invasiveness of nerve biopsies. Factors that play a role are microvascular changes in the endoneurium evoked by sepsis [16], which promotes vascular permeability and allows penetration of toxic factors into the nerve ends [17]. The endoneural edema resulting from increased permeability may impair energy delivery to the axon followed by axonal death. Direct toxic effects and mitochondrial dysfunction evoked by hyperglycemia may contribute to this process [18–20]. Animal experiments also identified channelopathy as a cause of rapidly occurring but reversible neuropathy [21]. Whether such a channelopathy also occurs in ICU patients, as a functional precursor of axonal degeneration that is found later in the course of critical illness, remains unclear [22].

In CIM, several factors are thought to play a role by negatively affecting muscle structure and function, all interacting in a complex manner [22]. A detailed overview of mediators and molecular mechanisms involved was published recently [23]. First, muscle atrophy can occur quite early during critical illness [24]. Muscle atrophy is brought about by increased breakdown and decreased synthesis of muscle protein [24, 25]. Several processes during critical illness may promote such wasting of muscle protein, which preferentially involves myosin [25, 26]. These include inflammation, immobilization, the endocrine stress responses, the rapidly developing nutritional deficit, impaired microcirculation, and denervation [22, 27, 28]. Key proinflammatory mediators involved include tumor necrosis factor alpha, interleukin-1, and interleukin-6 [23]. Recently, another stress-induced cytokine, growth and differentiation factor-15 (GDF-15), a member of the transforming growth factor beta super family, was identified as a mediator of muscle atrophy during critical illness [29]. In addition to muscle atrophy, other factors may contribute to decreased muscle function. Muscle membrane inexcitability induced by sodium channel dysfunction is an early finding in CIM [30] and predicts the development of clinical muscle weakness [31]. An altered intracellular calcium homeostasis, affecting the excitation–contraction coupling, has also been shown to contribute to reduced contractility in animal sepsis models [32, 33]. The muscles of septic patients show signs of bioenergetic failure, comprising oxidative stress, mitochondrial dysfunction, and ATP depletion [34]. Finally, muscle atrophy and weakness are not synonymous [22]. Indeed, muscle quality might be even more important than muscle mass in determining muscle function and the development of ICUAW [35]. Autophagy, which is a cellular housekeeping system that mediates removal of damaged large organelles and protein aggregates, is an important factor in maintaining muscle fiber integrity [36], and deficient autophagy may have a crucial role in the development of ICUAW [35].

## Risk factors

Several risk factors for the development of neuromuscular complications in the ICU have been reported. At the time when CIP was first described, sepsis, systemic inflammatory response syndrome (SIRS), and multiple organ failure (MOF) were considered to play a key role. Indeed, in the early case reports, sepsis was frequently present [2, 37]. Hence, subsequent studies specifically focused on patients with sepsis and organ failure and reported a high incidence of neuromuscular complications [38–40]. ICUAW was therefore considered to be the peripheral component of a general neuromuscular organ failure evoked by sepsis, with septic encephalopathy being the central component. An independent role for sepsis [35], bacteremia [10, 41], SIRS [40, 42], and MOF [8] was subsequently suggested by several prospective studies. Indirectly, a role for sepsis and MOF in the development of neuromuscular complications of critical illness was further supported when other independent risk factors were identified, such as the use of vasopressors [41, 43] or aminoglycosides [10], certain mediators of inflammation [44], and the presence of septic encephalopathy [39]. The risk associated with a higher severity of illness, as reflected by the Acute Physiology and Chronic Health Evaluation score, was demonstrated in several studies [10, 35, 42, 45]. Hyperglycemia was also found to be an independent risk factor for electrophysiological [38] and clinical [10] signs of ICUAW, and increasing insulin dose reduced signs of ICUAW [46]. These data offer the potential to prevent ICUAW by controlling glycemia with insulin (see ‘Prevention and therapy’). Providing early mobilization effectively reduced ICUAW [46], which is in line with the documented risk reduction in clinical or electrophysiological manifestations of ICUAW, associated with several indirect reflections of the duration of immobilization, such as duration of ICU stay [38, 43] and of mechanical ventilation prior to awakening [8], and the time until awakening and clinical assessment of muscle strength [35]. In one study, the duration of bed rest was the only factor consistently associated with weakness 3–24 months post ICU discharge [14]. This underscores the potential importance of treatments aiming to reduce the duration of immobilization in the ICU (see ‘Prevention and therapy’).

After a first case report of pronounced weakness in a patient treated with corticosteroids and neuromuscular blocking agents [47], multiple others followed. Denervated and steroid-treated animals showed muscle changes similar to those observed in critically ill patients [48]. Some prospective studies indeed suggested that corticosteroids [8, 35, 45] and (prolonged) neuromuscular blocking agents [18, 35, 39] may contribute to CIP/CIM or ICUAW. However, other prospective studies, of which some were randomized controlled trials, could not confirm such a role for corticosteroids [10, 13, 14, 18, 39, 40, 42–44, 46, 49] or for neuromuscular blocking agents [14, 50]. These apparently contradicting findings suggest that the relationship between these drugs and neuromuscular complications is more complex and depends on other factors such as dose, timing, and concomitant glycemic control [18]. Age was also identified as an independent risk factor for ICUAW [35, 46]. This may to some extent reflect the importance of the premorbid physiological muscle reserve, although the actual premorbid functional status is determined by many other factors. The premorbid functional status is often difficult to document due to the unplanned nature of ICU admissions. One report identified parenteral nutrition as a risk factor for CIP [39]. The effect of nutritional strategies as a modifiable factor is further discussed in ‘Prevention and therapy’. Other factors identified only in single studies might be incidental findings (female sex) [8] or related to other risk factors (hyperosmolality [39], hypoalbuminemia [38], renal replacement therapy [41]) and await confirmation.

Only few studies specifically addressed risk factors for respiratory weakness in the ICU. These studies demonstrated that respiratory muscle weakness is associated with infection or sepsis [51–53], disease severity [53], and peripheral weakness [51]. The phrenic nerve and diaphragm also show similar electrophysiologic abnormalities as the peripheral nerves and muscles [54], so these data suggest that respiratory weakness is indeed part of ICUAW [51, 53]. Additionally, the duration of mechanical ventilation may contribute to weakness [55, 56] and atrophy of the diaphragm [56, 57]. This time-dependent and early development of diaphragmatic atrophy and dysfunction is also labeled “ventilator-induced diaphragmatic dysfunction” (VIDD) [58].

## Clinical signs

ICUAW is characterized by symmetrical and flaccid weakness of the limbs, which is more pronounced in the proximal muscles than in the distal muscles [8, 59]. Facial and ocular muscles are often spared [60]. Patients with ICUAW therefore typically respond to a painful stimulus with facial grimacing but with no or minimal withdrawal of the limbs. Tendon reflexes are generally reduced, although these can be normal. In the case of coexistent CIP, sensory symptoms may be present, including reduced or absent sensitivity to pain, temperature, and vibration. However, examination of the sensory function is generally difficult in critically ill patients.

When ICUAW is present, the respiratory muscles are often affected [51]. This contributes to delayed weaning from mechanical ventilation, which is often the clinical problem with which these patients present.

### Small fiber neuropathy and autonomic dysfunction: different entities or part of the spectrum of neuromuscular failure in critically ill patients?

#### Small fiber neuropathy

In addition to CIP, which is a length-dependent large fiber neuropathy, several small series of well-selected patients demonstrated the presence of nonlength-dependent small fiber neuropathy on skin biopsies taken in the acute phase of critical illness [61, 62]. This degeneration or loss of small fibers may be responsible for neuropathic pain, stocking and glove sensory loss, numbness, cool extremities, and burning pain in survivors of critical illness [61, 63]. The available data indicate co-occurrence of small fiber neuropathy with CIP/CIM in a substantial number of cases, as well as a high incidence of sepsis or MOF in these patients. This suggests involvement of similar pathophysiological mechanisms [61]. Small fiber neuropathy, however, is also reported in patients without sepsis, MOF or CIP/CIM [61] and its pathophysiology has not yet been studied.

#### Autonomic dysfunction

Autonomic dysfunction, involving the peripheral and central sympathetic and parasympathetic systems, frequently occurs in critically ill patients [61, 64–66]. This dysfunction is documented by abnormal R-R variation, the cold face test, denervation of skin sweat glands on biopsy, and the skin wrinkle test. Because autonomic dysfunction is reported in patients with CIP/CIM, with reduced intra-epidermal nerve fiber density and septic encephalopathy, this again suggests a common pathway. Axonal degeneration of the sympathetic chain and vagal nerve was indeed documented on autopsy of patients with CIP [37]. Wieske et al. [64], however, did not find an association between abnormal heart rate variability, actually present in all studied patients, and ICUAW. Many confounders in the ICU affect heart rate variability and therefore this is possibly not the optimal indicator of autonomic dysfunction in this setting [64]. The impact of autonomic dysfunction on outcome also remains to be studied.

## Diagnosis

### Clinical evaluation of muscle strength

Current guidelines recommend a clinical diagnosis of ICUAW, made by bedside evaluation of the muscle strength with the use of the Medical Research Council (MRC) sum score [67]. This score appoints a value between 0 (no contraction at all) and 5 (normal muscle strength) for each of 12 muscle groups, including shoulder abduction, elbow flexion, wrist extension, hip flexion, knee extension, and dorsiflexion of the ankle, all scored bilaterally (Table 1). The sum score ranges between 0 and 60, and ICUAW is diagnosed with a total <48 [8]. Additional electrophysiological examination for clinical purposes is only indicated when the clinical presentation or evolution is atypical or when focal deficit is present [67]. The MRC sum score has a number of limitations. It does not detect the cause of weakness and cannot differentiate between CIP and CIM. Also, patients need to be awake and fully cooperative; a subset of patients, ranging between 10 % [13] and 75 % [68], do not fulfill this criterion during their ICU stay. Reproducibility in critically ill patients was reported to be good [6, 59], but this was not confirmed by other studies [68, 69]. Practical details of the scoring system concerning the adaptation of the original score for bedridden patients and use of rigorous criteria to define cooperation may be crucial to obtain reliable and reproducible results [70, 71]. The MRC sum score also has a ceiling effect and actual differentiation between 4 (subnormal strength) and 5 (normal strength) may be subjective [72].

**Table 1:** Medical Research Council sum score

Muscle group evaluated
Wrist extension
Elbow flexion
Shoulder abduction
Dorsiflexion foot
Knee extension
Hip flexion
Appointed score
0, no visible/palpable contraction
1, visible/palpable contraction without movement of the limb
2, movement of the limb, but not against gravity
3, movement against gravity
4, movement against gravity and some resistance
5, normal

Some other potentially interesting methods to evaluate the neuromuscular function include the use of handheld dynamometry and handgrip strength. Both are reproducible in the ICU [73]. Preliminary data suggest that handgrip strength relates to outcome [6] but these data need further confirmation. Muscle ultrasound allows detection of atrophy [24, 74] as well as architectural changes and fasciculation [75]. Ultrasound findings in the ICU setting, in contrast to other settings [76, 77], did not, however, correlate with muscle function or outcomes [75] in the few studies that are available. Preliminary data showed that reduced abductor polices strength following ulnar nerve stimulation in critically ill patients [78] but the relationship with global strength or outcome was not studied.

Respiratory muscle strength can be measured in the ICU using the maximal inspiratory pressure recorded following a volitional maneuver starting from the functional residual capacity [79]. This may be difficult, however, in ICU patients who are not cooperative. For such patients, a nonvolitional method using a one-way valve is a good alternative [80]. More sophisticated methods, such as measuring transdiaphragmatic pressure following magnetic phrenic nerve stimulation, is cumbersome and therefore not suited to daily practice [55].

### Electrophysiological testing

Because of the shortcomings of the MRC sum score, the interest in alternative diagnostic tools remains. Full electrophysiological testing, including nerve conduction studies (NCS) and electromyography (EMG), is cumbersome, not readily available in many ICUs, time consuming, and costly. NCS typically reveal reduced compound muscle action potentials (CMAPs); in the case of (coexistent) CIP, sensory nerve action potentials (SNAPs) may be reduced, and nerve conduction velocity is normal or only slightly reduced [81]. In both CIP and CIM, spontaneous electrical activity may be present on EMG. SNAPs in critically ill patients may also be reduced due to edema. Voluntary muscle contraction, which requires a cooperative patient, or alternatively more complex electrophysiological methods such as direct muscle stimulation can be used to differentiate between CIP and CIM [31, 82]. Simplified electrophysiological screening using the peroneal nerve CMAP, potentially supplemented by the sural nerve amplitude, may be used to identify patients who require more extensive testing [83, 84]. However, correlation between electrophysiological abnormalities and muscle weakness has not been studied in large patient populations; in particular, the importance of electrophysiological abnormalities in the absence of weakness is unclear.

### Biomarkers

The use of biomarkers could allow better targeting of future novel therapies. Creatine kinase may be increased in patients with ICUAW but is not a good biomarker [8]. Plasma levels of neurofilaments, which are biomarkers of axonal injury, are also elevated in patients with ICUAW [85]. Peak neurofilament levels showed good discriminative power for weakness but this peak only occurred after patients were clinically evaluable and therefore did not allow early diagnosis [85]. Although no validated biomarkers are available currently, identification of new mediators involved in the development of ICUAW, such as GDF-15, may represent promising future candidates.

## Outcomes of ICUAW

### Short-term implications

ICUAW is associated with prolonged ICU and hospital stay, prolonged duration of mechanical ventilation, and increased ICU and hospital mortality [6–8, 10, 51, 86]. Several mechanisms may explain increased morbidity and mortality, including associated respiratory muscle weakness [51], pharyngeal dysfunction, and symptomatic aspiration [9]. The association between weakness and poor outcomes could indicate that ICUAW is either a marker or a mediator of poor outcome. A randomized interventional study, which is the state of the art for examining causality, is evidently not possible for weakness. Propensity score matching provides the closest alternative to explore a potential causal relationship [87]. This involves matching of weak patients to not-weak patients for all risk factors that could contribute both to weakness and to poor outcomes [11]. In such a matched population, the time to live weaning from mechanical ventilation and the time to live ICU and hospital discharge were significantly longer in weak patients, as compared with matched not-weak patients. Additionally, healthcare-related hospitalization costs were 30.5 % higher. These data support a causal role between ICUAW and poor outcomes.

### Recovery from ICUAW and implications after the hospitalization phase

Recovery from weakness typically occurs within weeks or months [88], but the most severe cases may not recover. A systematic review indicated that severe sequellae were present in 28 % of patients [89]. Results from this thorough review were limited, however, by the lack of studies that included a large enough number of patients with sufficiently long follow-up. More recently, the proportion of patients with weakness was prospectively studied in patients with ARDS and showed a progressive decline over time from 36 % at hospital discharge to 22 % at 3 months, 7–15 % at 6 months, 4–14 % at 1 year and 9 % at 2 years [14, 90]. These data support that the majority of patients recover within months, reaching a plateau at around 1 year. Not surprisingly, ICUAW independently predicted the discharge destination [11]. Increasing evidence from several small prospective studies pointed to worse prognosis of CIP than of CIM [91–94]. CIM might recover faster and more completely whereas (coexistent) CIP possibly impedes recovery.

The actual impact of ICUAW on physical function and health-related quality of life was first addressed in a landmark study of ARDS survivors showing that both were significantly impaired up to 5 years after ICU admission [95, 96]. As generalized weakness and fatigue were the dominant prevailing complaints in these survivors, ICUAW was considered to be a crucial factor explaining the findings [96]. Several other studies have confirmed such persisting physical limitations [97–101] as well as reduced quality of life following ARDS, sepsis, and mechanical ventilation [97, 98, 102], in addition to impaired return to work [92, 95, 96] and cognitive dysfunction [103]. ARDS survivors with weakness at any time during the 2-year follow-up had reduced physical function and health-related quality of life as compared with patients without weakness [97], although the independent contribution of ICUAW to these outcomes has not yet been studied. In contrast, weakness did independently contribute to 1-year mortality [11]. Additionally, the persistence and higher severity of weakness at ICU discharge further increased the risk of death within the first year [11].

Finally, although the number of patients with persisting weakness is small, there is a marked reduction in physical function in the majority of ARDS survivors [95]. This suggests that weakness is not the only culprit limiting physical function. Other potentially relevant factors in addition to muscle strength include proprioception, gait balance, spatial attention, cognitive function, mental health, central nervous dysfunction, pain, and entrapment neuropathy [63, 92, 97, 104].

## Prevention and therapy

Aggressive treatment of sepsis is considered to be a cornerstone in the prevention of ICUAW. Although anti-inflammatory therapies in sepsis in general have been disappointing and specific neuromuscular effects have not been studied, targeting newly discovered inflammatory mediators involved in muscle atrophy, such as GDF-15, may open new perspectives. Further preventive strategies include the reduction or avoidance of other known risk factors but only few randomized trials addressing these are available.

Insulin treatment aimed at normalizing glycemia as compared with tolerating hyperglycemia up to the renal threshold significantly reduced the incidence of electrophysiological signs of CIP/CIM and the need for prolonged mechanical ventilation in long-stay medical [18] and surgical [43] ICU patients. A subsequent multicenter trial [105] found increased mortality in patients treated to strict normoglycemia as compared with those receiving insulin to target somewhat higher blood glucose levels. Consequently, the optimal target for blood glucose remains a matter of debate and ongoing efforts are being made to more safely control glucose in the ICU [106].

Reducing the duration of immobilization is another potentially important target in the prevention of ICUAW. This target can be realized by decreasing the levels of sedation. Approaches aimed at sedating patients to the minimal level needed for comfort and safety clearly have overall beneficial effects [107]. The actual impact of such strategies on ICUAW has not been studied. Reducing the duration of immobilization with early physical therapy adjusted to the medical situation, muscle strength, and level of cooperation is another strategy that was shown to be safe and feasible in the ICU and improved outcome [108]. Additionally, quadriceps strength at hospital discharge improved in long-stay patients receiving passive or active exercise training using a bedside ergometer, as compared with patients receiving standard physiotherapy [109]. This training involved 20 minutes of bedcyling 5 days per week, from the 5th day following ICU admission. Both functional status and health-related quality of life at hospital discharge were also improved [109]. Early mobilization and occupational therapy within 72 hours of initiation of mechanical ventilation in addition to daily sedation interruption improved outcome, including functional status, although the incidence of ICUAW was not reduced [110]. The training consisted of an individually tailored daily program starting with passive range-of-motion exercises in unresponsive patients, progressing to active range-of-motion exercises, bed mobility, sitting upright, transfer training, and eventually walking. A secondary analysis of this trial reported that, after correction of other risk factors, both early mobilization and increasing insulin dose independently prevented ICUAW [46]. Despite clear benefits from early mobilization of patients in the ICU, daily practice appears to vary significantly among centers and clinical settings [111, 112] and many barriers exist, hampering a wide implementation. Identification of subgroups of patients who are most likely to benefit would allow a better allocation of resources [113]. Patients with ICUAW might represent a group of particular interest but data supporting such an approach are currently lacking [114]. Analogous to the peripheral muscles, early mobilization of the diaphragm allowing for spontaneous breathing while avoiding overactivation is considered standard practice to minimize respiratory muscle weakness [56, 115].

A substantial number of ICU patients are not able to participate in active mobilization early. In this population, electrical muscle stimulation (EMS) could theoretically be used to activate and cultivate muscles during this phase. Several studies suggest potential beneficial effects of EMS in critically ill patients. The evidence remains inconclusive, however, because of equivocal results and several methodological issues [116, 117].

Finally, malnutrition was originally considered a major contributor to ICUAW and correction of the nutritional deficit using parenteral feeding appeared evident [2]. This paradigm was recently challenged by Puthacheary et al. [24], who reported that increased protein delivery during the first week in the ICU was associated with more pronounced muscle wasting. In addition, avoiding parenteral nutrition during the first week in the ICU, as compared with early supplementation of deficient enteral feeding, reduced the incidence of weakness and enhanced recovery thereof [35]. This beneficial effect was explained by enhanced autophagic quality control in the muscle fibers, whereas markers of atrophy remained unaffected. These findings suggest that the early catabolic phase during critical illness cannot be averted by artificial nutrition. Certain catabolic pathways, in particular autophagy, may be crucial to maintain muscle quality and function. Hence, targeting autophagy may be worthwhile to investigate further as a future treatment strategy. Also, early “trophic” feeding via the enteral route instead of full enteral feeding during the first week in the ICU did not have detrimental effects on physical function or strength measurements up to 1 year following ARDS [98].

## Conclusions

ICUAW affects many critically ill patients and is associated with prolonged duration of mechanical ventilation, ICU stay, and hospital stay, as well as increased ICU and hospital mortality and healthcare-related hospitalization costs. In addition, following the acute phase of illness, ICUAW may be an important contributor to the physical limitations persisting in ICU survivors, associated with reduced health-related quality of life, as well as higher 1-year mortality. This underscores the importance for future research on prevention of ICUAW. In parallel, the underlying mechanisms contributing to the poor outcome of weak patients and the potential to avert unfavorable outcomes by post-ICU follow-up of these patients should also be investigated further.

## Abbreviations

ARDS: Acute respiratory distress syndrome
CIM: Critical illness myopathy
CIP: Critical illness polyneuropathy
CMAP: Compound muscle action potential
EMG: Electromyography
EMS: Electrical muscle stimulation
GDF-15: Growth and differentiation factor-15
ICUAW: Intensive care unit acquired weakness
MOF: Multiple organ failure
MRC: Medical Research Council
NCS: Nerve conduction studies
PICS: Post intensive care syndrome
SIRS: Systemic inflammatory response syndrome
SNAP: Sensory nerve action potential
VIDD: Ventilator-induced diaphragmatic dysfunction
